# Poly[(μ_4_-benzene-1,3,5-tricarboxyl­ato)bis­(*N*,*N*-dimethyl­acetamide)­terbium(III)]

**DOI:** 10.1107/S1600536812010264

**Published:** 2012-03-14

**Authors:** Kun Liu

**Affiliations:** a The Department of Physics-Chemistry, Henan Polytechnic University, Jiaozuo 454000, People’s Republic of China

## Abstract

The title compound, [Tb(C_9_H_3_O_6_)(C_4_H_9_NO)_2_], shows a rare-earth three-dimensional metal-organic framework structure. In this complex of an eight-coordinated Tb^3+^ ion, the asymmetric unit contains one benzene-1,3,5-tricarb­oxy­lic ligand and two coordinated dimethyl­acetamide mol­ecules. Each Tb^3+^ ion is coordinated by six O atoms from four carboxyl­ate groups of the benzene-1,3,5-tricarb­oxy­lic ligands and two O atoms from two terminal dimethyl­acetamide mol­ecules.

## Related literature

For metal-organic framework compounds with adsorption, catalytic and fluorescence properties, see: Sun *et al.* (2006 ▶); Ravon *et al.* (2008 ▶); Allendorf *et al.* (2009 ▶). For isotypic rare earth complexes, see: Thirumurugan & Natarajan (2004 ▶) and for rare earth coordination polymers, see: Guo *et al.* (2006 ▶).
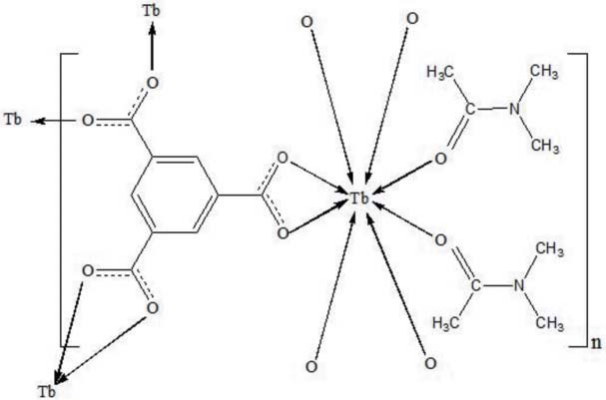


## Experimental

### 

#### Crystal data

[Tb(C_9_H_3_O_6_)(C_4_H_9_NO)_2_]
*M* *_r_* = 540.28
Monoclinic, 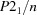

*a* = 10.8924 (6) Å
*b* = 16.7740 (9) Å
*c* = 10.9631 (6) Å
β = 102.254 (1)°
*V* = 1957.42 (18) Å^3^
*Z* = 4
Mo *K*α radiation
μ = 3.66 mm^−1^
*T* = 273 K
0.60 × 0.40 × 0.40 mm

#### Data collection

Bruker APEXII CCD diffractometer
Absorption correction: multi-scan (*SADABS*; Sheldrick, 2008*a* ▶) *T*_min_ = 0.218, *T*_max_ = 0.322
10235 measured reflections
3433 independent reflections
2385 reflections with *I* > 2σ(*I*)
*R*_int_ = 0.055

#### Refinement

*R*[*F* ^2^ > 2σ(*F* ^2^)] = 0.034
*wR*(*F* ^2^) = 0.066
*S* = 0.89
3433 reflections
259 parameters
24 restraints
H-atom parameters constrained
Δρ_max_ = 1.52 e Å^−3^
Δρ_min_ = −0.79 e Å^−3^

### 

Data collection: *APEX2* (Bruker, 2004 ▶); cell refinement: *SAINT* (Bruker, 2004 ▶); data reduction: *SAINT*; program(s) used to solve structure: *SHELXS97* (Sheldrick, 2008*b* ▶); program(s) used to refine structure: *SHELXL97* (Sheldrick, 2008*b* ▶); molecular graphics: *SHELXTL* (Sheldrick, 2008*b* ▶); software used to prepare material for publication: *SHELXTL*.

## Supplementary Material

Crystal structure: contains datablock(s) I, global. DOI: 10.1107/S1600536812010264/go2045sup1.cif

Structure factors: contains datablock(s) I. DOI: 10.1107/S1600536812010264/go2045Isup2.hkl

Additional supplementary materials:  crystallographic information; 3D view; checkCIF report

## Figures and Tables

**Fig. 1.**
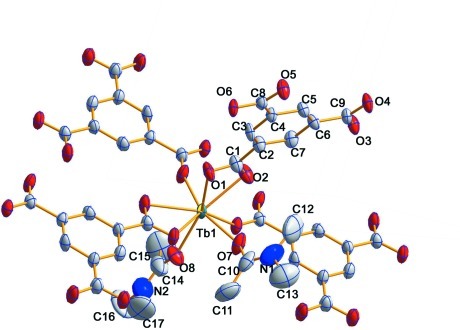
Coordination environment of Tb in the complex with hydrogen atoms removed for the clarity. Displacement ellipsoids are drawn at the 30% probability level.

## supplementary crystallographic information

### Comment

Metal-organic framework design and construction is currently a flourishing field of research owing to the intriguing molecular topologies and the potentially exploitable adsorption (Sun *et al.*, 2006), catalytic (Ravon *et al.*, 2008) and fluorescence (Allendorf *et al.*, 2009) properties of these types of compounds. As functional metal centers, rare earth metals are attracting more attention from synthetic chemists for their unusual coordination properties and special chemical characteristics arising from interactions with the 4f electrons and the propensity to form isostructural complexes (Thirumurugan *et al.*, 2004). Many coordination polymers utilizing the rare earth elements have been synthesized (Guo *et al.*, 2006). The title compound shows a rare-earth three-dimensional metal-organic framework structure. In this complex of an eight-coordinated Tb^3+^ ion, the asymmetric unit contains one benzene-1,3,5-tricarboxylic ligand and two coordinated dimethylacetamide molecules.

Each Tb^3+^ is coordinated with six oxygen atoms from four carboxylate groups of the benzene-1,3,5-tricarboxylic ligands and two oxygen atoms from two terminal dimethylacetamide molecules, (Figure 1).

### Experimental

All reagents were of analytical grade. A mixture of terbium nitrate (40 mg, 0.10 mmol) and benzene-*1,3,5*-tricarboxylate acid (10 mg, 0.05 mmol) was dissolved in *N,N'*-dimethylacetamide (25 ml) at room temperature. This mixture was placed at 60 °C for 3 days giving rise to colourless rod crystals.

### Refinement

H atoms bound to C atoms were placed in calculated positions and treated as riding on their parent atoms, with C—H = 0.93 Å (aromatic C), C—H = 0.96 Å (methly C) and with *U*_iso_(H) = 1.2Ueq(C). ISOR restraints were placed on atoms C13 N1 N2 and C14. The position of all methyl hydrogens was checked on a final difference map and shown to be satisfactory.

### Crystal data

**Table:**

[Tb(C_9_H_3_O_6_)(C_4_H_9_NO)_2_]	*F*(000) = 1064
*M**_r_* = 540.28	*D*_x_ = 1.833 Mg m^−^^3^
Monoclinic, *P*2_1_/*n*	Mo *K*α radiation, λ = 0.71073 Å
*a* = 10.8924 (6) Å	Cell parameters from 2246 reflections
*b* = 16.7740 (9) Å	θ = 2.3–22.4°
*c* = 10.9631 (6) Å	µ = 3.66 mm^−^^1^
β = 102.254 (1)°	*T* = 273 K
*V* = 1957.42 (18) Å^3^	Rod, colourless
*Z* = 4	0.60 × 0.40 × 0.40 mm

### Data collection

**Table:**

Bruker APEXII CCD diffractometer	3433 independent reflections
Radiation source: fine-focus sealed tube	2385 reflections with *I* > 2σ(*I*)
Graphite monochromator	*R*_int_ = 0.055
Detector resolution: 8.33 pixels mm^-1^	θ_max_ = 25.0°, θ_min_ = 2.3°
phi and ω scans	*h* = −12→12
Absorption correction: multi-scan (*SADABS*; Sheldrick, 2008*a*)	*k* = −17→19
*T*_min_ = 0.218, *T*_max_ = 0.322	*l* = −13→13
10235 measured reflections	

### Refinement

**Table:**

Refinement on *F*^2^	Primary atom site location: structure-invariant direct methods
Least-squares matrix: full	Secondary atom site location: difference Fourier map
*R*[*F*^2^ > 2σ(*F*^2^)] = 0.034	Hydrogen site location: inferred from neighbouring sites
*wR*(*F*^2^) = 0.066	H-atom parameters constrained
*S* = 0.89	*w* = 1/[σ^2^(*F*_o_^2^) + (0.0188*P*)^2^] where *P* = (*F*_o_^2^ + 2*F*_c_^2^)/3
3433 reflections	(Δ/σ)_max_ = 0.002
259 parameters	Δρ_max_ = 1.52 e Å^−^^3^
24 restraints	Δρ_min_ = −0.79 e Å^−^^3^

### Special details

**Table:**

Geometry. All e.s.d.'s (except the e.s.d. in the dihedral angle between two l.s. planes) are estimated using the full covariance matrix. The cell e.s.d.'s are taken into account individually in the estimation of e.s.d.'s in distances, angles and torsion angles; correlations between e.s.d.'s in cell parameters are only used when they are defined by crystal symmetry. An approximate (isotropic) treatment of cell e.s.d.'s is used for estimating e.s.d.'s involving l.s. planes.
Refinement. Refinement of *F*^2^ against ALL reflections. The weighted *R*-factor *wR* and goodness of fit *S* are based on *F*^2^, conventional *R*-factors *R* are based on *F*, with *F* set to zero for negative *F*^2^. The threshold expression of *F*^2^ > σ(*F*^2^) is used only for calculating *R*-factors(gt) *etc*. and is not relevant to the choice of reflections for refinement. *R*-factors based on *F*^2^ are statistically about twice as large as those based on *F*, and *R*- factors based on ALL data will be even larger.

### Fractional atomic coordinates and isotropic or equivalent isotropic displacement parameters (Å^2^)

**Table:**

	*x*	*y*	*z*	*U*_iso_*/*U*_eq_	
Tb1	0.65099 (3)	0.112365 (17)	−0.08321 (3)	0.02780 (11)	
C1	0.6570 (6)	0.2120 (4)	0.1214 (6)	0.0390 (17)	
C2	0.6452 (6)	0.2784 (4)	0.2122 (6)	0.0344 (16)	
C3	0.7451 (6)	0.3279 (3)	0.2632 (6)	0.0337 (16)	
H3	0.8227	0.3194	0.2428	0.040*	
C4	0.7325 (5)	0.3896 (4)	0.3438 (5)	0.0283 (14)	
C5	0.6154 (5)	0.4017 (3)	0.3746 (5)	0.0326 (16)	
H5	0.6065	0.4423	0.4298	0.039*	
C6	0.5112 (6)	0.3539 (4)	0.3241 (6)	0.0324 (16)	
C7	0.5287 (6)	0.2932 (4)	0.2434 (6)	0.0372 (17)	
H7	0.4607	0.2610	0.2087	0.045*	
C8	0.8415 (6)	0.4454 (4)	0.3953 (6)	0.0311 (15)	
C9	0.3875 (6)	0.3652 (4)	0.3574 (6)	0.0303 (16)	
C10	0.5469 (7)	0.2846 (5)	−0.2411 (8)	0.062 (2)	
C11	0.5890 (9)	0.2864 (5)	−0.3628 (8)	0.097 (3)	
H11A	0.6323	0.2377	−0.3727	0.145*	
H11B	0.6446	0.3307	−0.3633	0.145*	
H11C	0.5172	0.2918	−0.4302	0.145*	
C12	0.4520 (11)	0.3475 (6)	−0.0923 (11)	0.138 (5)	
H12A	0.4480	0.2935	−0.0647	0.208*	
H12B	0.3688	0.3694	−0.1135	0.208*	
H12C	0.5023	0.3786	−0.0267	0.208*	
C13	0.4954 (11)	0.4249 (6)	−0.2661 (10)	0.131 (4)	
H13A	0.5156	0.4176	−0.3464	0.196*	
H13B	0.5519	0.4630	−0.2189	0.196*	
H13C	0.4107	0.4440	−0.2767	0.196*	
C14	0.6376 (9)	−0.0207 (8)	−0.3242 (9)	0.094 (4)	
C15	0.5893 (11)	−0.0955 (5)	−0.2590 (11)	0.131 (5)	
H15A	0.6059	−0.1434	−0.3006	0.197*	
H15B	0.5005	−0.0907	−0.2640	0.197*	
H15C	0.6321	−0.0977	−0.1730	0.197*	
C16	0.6552 (11)	−0.1129 (6)	−0.4926 (10)	0.140 (5)	
H16A	0.6042	−0.1519	−0.4627	0.211*	
H16B	0.7388	−0.1334	−0.4849	0.211*	
H16C	0.6200	−0.1013	−0.5787	0.211*	
C17	0.7101 (10)	0.0352 (7)	−0.4836 (10)	0.141 (5)	
H17A	0.6452	0.0544	−0.5505	0.211*	
H17B	0.7820	0.0201	−0.5159	0.211*	
H17C	0.7332	0.0764	−0.4224	0.211*	
N1	0.5073 (8)	0.3492 (5)	−0.1998 (8)	0.091 (2)	
N2	0.6593 (8)	−0.0410 (5)	−0.4200 (8)	0.098 (3)	
O1	0.7562 (4)	0.2041 (3)	0.0827 (4)	0.0467 (13)	
O2	0.5632 (4)	0.1690 (3)	0.0810 (4)	0.0504 (13)	
O3	0.3021 (4)	0.3156 (2)	0.3202 (4)	0.0395 (12)	
O4	0.3703 (4)	0.4242 (2)	0.4224 (4)	0.0410 (12)	
O5	0.8182 (4)	0.5022 (2)	0.4600 (4)	0.0447 (12)	
O6	0.9428 (4)	0.4319 (2)	0.3650 (4)	0.0344 (11)	
O7	0.5483 (4)	0.2215 (3)	−0.1834 (5)	0.0544 (14)	
O8	0.6406 (5)	0.0438 (3)	−0.2719 (5)	0.0551 (14)	

### Atomic displacement parameters (Å^2^)

**Table:**

	*U*^11^	*U*^22^	*U*^33^	*U*^12^	*U*^13^	*U*^23^
Tb1	0.01970 (16)	0.02548 (17)	0.04074 (19)	0.00104 (17)	0.01207 (13)	0.00197 (17)
C1	0.034 (4)	0.037 (4)	0.049 (5)	−0.005 (3)	0.016 (4)	−0.011 (3)
C2	0.026 (4)	0.033 (4)	0.046 (4)	−0.007 (3)	0.011 (3)	−0.009 (3)
C3	0.023 (4)	0.031 (4)	0.052 (4)	0.000 (3)	0.020 (3)	−0.007 (3)
C4	0.022 (3)	0.028 (3)	0.036 (4)	0.000 (3)	0.007 (3)	−0.003 (3)
C5	0.030 (4)	0.033 (4)	0.038 (4)	0.001 (3)	0.015 (3)	−0.006 (3)
C6	0.029 (4)	0.033 (4)	0.039 (4)	−0.006 (3)	0.016 (3)	−0.007 (3)
C7	0.028 (4)	0.034 (4)	0.049 (4)	−0.009 (3)	0.009 (3)	−0.007 (3)
C8	0.023 (4)	0.035 (4)	0.036 (4)	0.002 (3)	0.009 (3)	0.001 (3)
C9	0.025 (4)	0.029 (4)	0.040 (4)	0.003 (3)	0.013 (3)	−0.003 (3)
C10	0.036 (5)	0.059 (6)	0.078 (7)	0.003 (4)	−0.014 (5)	−0.001 (5)
C11	0.092 (8)	0.111 (8)	0.079 (7)	0.000 (6)	−0.002 (6)	0.046 (6)
C12	0.152 (12)	0.108 (9)	0.145 (11)	0.077 (8)	0.008 (10)	−0.025 (8)
C13	0.145 (6)	0.109 (5)	0.126 (6)	−0.013 (4)	0.001 (4)	0.011 (4)
C14	0.058 (6)	0.152 (10)	0.068 (7)	0.016 (7)	0.005 (6)	−0.041 (7)
C15	0.163 (13)	0.065 (8)	0.179 (12)	−0.028 (7)	0.066 (10)	0.019 (7)
C16	0.156 (12)	0.139 (10)	0.125 (9)	0.008 (9)	0.027 (9)	−0.099 (8)
C17	0.126 (11)	0.195 (13)	0.117 (10)	−0.008 (9)	0.058 (9)	0.065 (9)
N1	0.099 (5)	0.066 (4)	0.095 (4)	0.008 (4)	−0.011 (4)	0.006 (4)
N2	0.093 (5)	0.103 (5)	0.094 (4)	0.015 (4)	0.013 (4)	−0.025 (4)
O1	0.025 (3)	0.058 (3)	0.062 (3)	−0.006 (2)	0.021 (3)	−0.025 (2)
O2	0.041 (3)	0.053 (3)	0.063 (3)	−0.018 (3)	0.025 (3)	−0.029 (3)
O3	0.026 (3)	0.036 (3)	0.061 (3)	−0.008 (2)	0.019 (2)	−0.016 (2)
O4	0.024 (3)	0.040 (3)	0.064 (3)	−0.004 (2)	0.022 (2)	−0.014 (2)
O5	0.026 (3)	0.041 (3)	0.068 (3)	−0.009 (2)	0.014 (3)	−0.028 (2)
O6	0.018 (2)	0.032 (3)	0.054 (3)	0.003 (2)	0.010 (2)	−0.006 (2)
O7	0.034 (3)	0.037 (3)	0.095 (4)	0.007 (2)	0.020 (3)	0.032 (3)
O8	0.055 (3)	0.054 (3)	0.062 (4)	−0.014 (3)	0.024 (3)	−0.026 (3)

### Geometric parameters (Å, º)

**Table:**

Tb1—O5^i^	2.271 (4)	C11—H11A	0.9600
Tb1—O7	2.299 (4)	C11—H11B	0.9600
Tb1—O6^ii^	2.339 (4)	C11—H11C	0.9600
Tb1—O8	2.348 (5)	C12—N1	1.433 (11)
Tb1—O2	2.406 (4)	C12—H12A	0.9600
Tb1—O4^iii^	2.454 (4)	C12—H12B	0.9600
Tb1—O3^iii^	2.456 (4)	C12—H12C	0.9600
Tb1—O1	2.471 (4)	C13—N1	1.454 (10)
C1—O1	1.248 (7)	C13—H13A	0.9600
C1—O2	1.253 (7)	C13—H13B	0.9600
C1—C2	1.517 (8)	C13—H13C	0.9600
C2—C3	1.388 (8)	C14—N2	1.175 (10)
C2—C7	1.405 (8)	C14—O8	1.221 (11)
C3—C4	1.387 (7)	C14—C15	1.589 (13)
C3—H3	0.9300	C15—H15A	0.9600
C4—C5	1.402 (7)	C15—H15B	0.9600
C4—C8	1.523 (8)	C15—H15C	0.9600
C5—C6	1.403 (8)	C16—N2	1.440 (10)
C5—H5	0.9300	C16—H16A	0.9600
C6—C7	1.389 (8)	C16—H16B	0.9600
C6—C9	1.481 (8)	C16—H16C	0.9600
C7—H7	0.9300	C17—N2	1.609 (11)
C8—O6	1.238 (6)	C17—H17A	0.9600
C8—O5	1.246 (6)	C17—H17B	0.9600
C9—O3	1.250 (7)	C17—H17C	0.9600
C9—O4	1.257 (6)	O3—Tb1^iv^	2.456 (4)
C10—O7	1.232 (9)	O4—Tb1^iv^	2.454 (4)
C10—N1	1.285 (10)	O5—Tb1^v^	2.271 (4)
C10—C11	1.500 (11)	O6—Tb1^vi^	2.338 (4)
			
O5^i^—Tb1—O7	158.01 (15)	C6—C9—Tb1^iv^	179.1 (5)
O5^i^—Tb1—O6^ii^	84.24 (14)	O7—C10—N1	120.8 (9)
O7—Tb1—O6^ii^	77.77 (15)	O7—C10—C11	120.1 (8)
O5^i^—Tb1—O8	95.69 (17)	N1—C10—C11	119.1 (9)
O7—Tb1—O8	92.37 (18)	C10—C11—H11A	109.5
O6^ii^—Tb1—O8	76.59 (15)	C10—C11—H11B	109.5
O5^i^—Tb1—O2	84.73 (16)	H11A—C11—H11B	109.5
O7—Tb1—O2	79.21 (17)	C10—C11—H11C	109.5
O6^ii^—Tb1—O2	77.74 (14)	H11A—C11—H11C	109.5
O8—Tb1—O2	154.14 (16)	H11B—C11—H11C	109.5
O5^i^—Tb1—O4^iii^	76.21 (14)	N1—C12—H12A	109.5
O7—Tb1—O4^iii^	125.67 (15)	N1—C12—H12B	109.5
O6^ii^—Tb1—O4^iii^	144.63 (14)	H12A—C12—H12B	109.5
O8—Tb1—O4^iii^	76.37 (16)	N1—C12—H12C	109.5
O2—Tb1—O4^iii^	128.27 (16)	H12A—C12—H12C	109.5
O5^i^—Tb1—O3^iii^	128.97 (14)	H12B—C12—H12C	109.5
O7—Tb1—O3^iii^	72.82 (14)	N1—C13—H13A	109.5
O6^ii^—Tb1—O3^iii^	139.07 (14)	N1—C13—H13B	109.5
O8—Tb1—O3^iii^	76.85 (15)	H13A—C13—H13B	109.5
O2—Tb1—O3^iii^	122.52 (15)	N1—C13—H13C	109.5
O4^iii^—Tb1—O3^iii^	52.85 (13)	H13A—C13—H13C	109.5
O5^i^—Tb1—O1	94.68 (16)	H13B—C13—H13C	109.5
O7—Tb1—O1	87.62 (17)	N2—C14—O8	133.4 (13)
O6^ii^—Tb1—O1	130.58 (13)	N2—C14—C15	108.7 (11)
O8—Tb1—O1	151.84 (15)	O8—C14—C15	117.8 (9)
O2—Tb1—O1	53.07 (14)	C14—C15—H15A	109.5
O4^iii^—Tb1—O1	80.81 (14)	C14—C15—H15B	109.5
O3^iii^—Tb1—O1	76.26 (14)	H15A—C15—H15B	109.5
O1—C1—O2	121.3 (6)	C14—C15—H15C	109.5
O1—C1—C2	120.0 (6)	H15A—C15—H15C	109.5
O2—C1—C2	118.5 (6)	H15B—C15—H15C	109.5
C3—C2—C7	117.8 (6)	N2—C16—H16A	109.5
C3—C2—C1	122.3 (5)	N2—C16—H16B	109.5
C7—C2—C1	119.8 (6)	H16A—C16—H16B	109.5
C4—C3—C2	121.9 (5)	N2—C16—H16C	109.5
C4—C3—H3	119.1	H16A—C16—H16C	109.5
C2—C3—H3	119.1	H16B—C16—H16C	109.5
C3—C4—C5	118.7 (6)	N2—C17—H17A	109.5
C3—C4—C8	121.3 (5)	N2—C17—H17B	109.5
C5—C4—C8	120.0 (5)	H17A—C17—H17B	109.5
C4—C5—C6	121.5 (5)	N2—C17—H17C	109.5
C4—C5—H5	119.2	H17A—C17—H17C	109.5
C6—C5—H5	119.2	H17B—C17—H17C	109.5
C7—C6—C5	117.5 (5)	C10—N1—C12	120.3 (9)
C7—C6—C9	120.4 (6)	C10—N1—C13	124.1 (10)
C5—C6—C9	122.1 (5)	C12—N1—C13	114.8 (9)
C6—C7—C2	122.6 (6)	C14—N2—C16	138.5 (11)
C6—C7—H7	118.7	C14—N2—C17	108.0 (10)
C2—C7—H7	118.7	C16—N2—C17	113.5 (9)
O6—C8—O5	126.4 (6)	C1—O1—Tb1	90.9 (4)
O6—C8—C4	117.2 (6)	C1—O2—Tb1	93.8 (4)
O5—C8—C4	116.3 (5)	C9—O3—Tb1^iv^	93.0 (3)
O3—C9—O4	121.3 (5)	C9—O4—Tb1^iv^	92.9 (4)
O3—C9—C6	119.2 (5)	C8—O5—Tb1^v^	159.8 (4)
O4—C9—C6	119.5 (6)	C8—O6—Tb1^vi^	150.0 (4)
O3—C9—Tb1^iv^	60.7 (3)	C10—O7—Tb1	151.8 (5)
O4—C9—Tb1^iv^	60.6 (3)	C14—O8—Tb1	147.0 (7)
			
O1—C1—C2—C3	−5.5 (11)	O2—Tb1—O1—C1	−5.1 (4)
O2—C1—C2—C3	178.8 (7)	O4^iii^—Tb1—O1—C1	−160.2 (4)
O1—C1—C2—C7	172.2 (6)	O3^iii^—Tb1—O1—C1	146.0 (4)
O2—C1—C2—C7	−3.6 (10)	O1—C1—O2—Tb1	−9.5 (8)
C7—C2—C3—C4	0.7 (10)	C2—C1—O2—Tb1	166.2 (5)
C1—C2—C3—C4	178.4 (6)	O5^i^—Tb1—O2—C1	105.0 (5)
C2—C3—C4—C5	0.3 (9)	O7—Tb1—O2—C1	−90.1 (5)
C2—C3—C4—C8	−177.6 (6)	O6^ii^—Tb1—O2—C1	−169.7 (5)
C3—C4—C5—C6	−1.2 (9)	O8—Tb1—O2—C1	−162.8 (4)
C8—C4—C5—C6	176.8 (6)	O4^iii^—Tb1—O2—C1	37.2 (5)
C4—C5—C6—C7	0.8 (9)	O3^iii^—Tb1—O2—C1	−28.6 (5)
C4—C5—C6—C9	179.0 (6)	O1—Tb1—O2—C1	5.1 (4)
C5—C6—C7—C2	0.3 (10)	O4—C9—O3—Tb1^iv^	1.1 (6)
C9—C6—C7—C2	−177.9 (6)	C6—C9—O3—Tb1^iv^	−179.0 (5)
C3—C2—C7—C6	−1.1 (10)	O3—C9—O4—Tb1^iv^	−1.1 (6)
C1—C2—C7—C6	−178.8 (6)	C6—C9—O4—Tb1^iv^	179.0 (5)
C3—C4—C8—O6	−2.1 (9)	O6—C8—O5—Tb1^v^	−6.1 (17)
C5—C4—C8—O6	180.0 (5)	C4—C8—O5—Tb1^v^	177.0 (9)
C3—C4—C8—O5	175.2 (6)	O5—C8—O6—Tb1^vi^	74.9 (11)
C5—C4—C8—O5	−2.7 (9)	C4—C8—O6—Tb1^vi^	−108.2 (8)
C7—C6—C9—O3	5.8 (9)	N1—C10—O7—Tb1	−122.4 (11)
C5—C6—C9—O3	−172.3 (6)	C11—C10—O7—Tb1	59.0 (15)
C7—C6—C9—O4	−174.3 (6)	O5^i^—Tb1—O7—C10	165.9 (11)
C5—C6—C9—O4	7.6 (9)	O6^ii^—Tb1—O7—C10	−158.3 (12)
O7—C10—N1—C12	−6.3 (13)	O8—Tb1—O7—C10	−82.5 (12)
C11—C10—N1—C12	172.2 (9)	O2—Tb1—O7—C10	122.1 (12)
O7—C10—N1—C13	−176.0 (8)	O4^iii^—Tb1—O7—C10	−7.6 (13)
C11—C10—N1—C13	2.6 (14)	O3^iii^—Tb1—O7—C10	−7.1 (12)
O8—C14—N2—C16	−177.2 (11)	O1—Tb1—O7—C10	69.3 (12)
C15—C14—N2—C16	−0.4 (18)	N2—C14—O8—Tb1	−160.2 (9)
O8—C14—N2—C17	4.1 (17)	C15—C14—O8—Tb1	23.3 (16)
C15—C14—N2—C17	−179.2 (8)	O5^i^—Tb1—O8—C14	6.7 (11)
O2—C1—O1—Tb1	9.3 (7)	O7—Tb1—O8—C14	−152.8 (11)
C2—C1—O1—Tb1	−166.4 (6)	O6^ii^—Tb1—O8—C14	−76.0 (11)
O5^i^—Tb1—O1—C1	−85.0 (4)	O2—Tb1—O8—C14	−83.0 (12)
O7—Tb1—O1—C1	73.1 (4)	O4^iii^—Tb1—O8—C14	81.0 (11)
O6^ii^—Tb1—O1—C1	1.5 (5)	O3^iii^—Tb1—O8—C14	135.4 (11)
O8—Tb1—O1—C1	163.7 (4)	O1—Tb1—O8—C14	117.8 (10)

Symmetry codes: (i) −*x*+3/2, *y*−1/2, −*z*+1/2; (ii) *x*−1/2, −*y*+1/2, *z*−1/2; (iii) *x*+1/2, −*y*+1/2, *z*−1/2; (iv) *x*−1/2, −*y*+1/2, *z*+1/2; (v) −*x*+3/2, *y*+1/2, −*z*+1/2; (vi) *x*+1/2, −*y*+1/2, *z*+1/2.
